# Supplementary material for: Mixed methods pilot evaluation of a gender-sensitivity training for HIV care providers in Uganda: Effects on providers and clients
Source: PLOS Glob Public Health. 2025 Sep 18;5(9):e0004247. doi: 10.1371/journal.pgph.0004247 (PMC12445538; doi:10.1371/journal.pgph.0004247)
Supplement: S1 Text — (DOCX) [file pgph.0004247.s001.docx]

**A mixed methods pilot evaluation of a gender sensitive training for HIV providers in central Uganda: preliminary intervention effects on HIV provider and client outcomes**

Supplemental Files

Table of Contents

[Table A. Covariates controlled for in client cohort models 1](#_Toc207898464)

[Table B. Statistics for the time by intervention by gender interaction on competence to provide gender-sensitive care among the HIV provider cohort 2](#_Toc207898465)

[Table C. Statistics for the time by intervention by cadre interaction on competence to provide gender-sensitive care among the HIV provider cohort 4](#_Toc207898466)

[Figure A. Depiction of the time by intervention by gender interaction on perceived participatory decision-making with HIV providers among the client cohort 5](#_Toc207898467)

[Table D. Statistics for the time by intervention by gender interaction on perceived participatory decision-making with HIV providers among the client cohort 6](#_Toc207898468)

[Table E. CONSORT 2010 checklist of information to include when reporting a pilot or feasibility trial 7](#_Toc207898469)

# Table A. Covariates controlled for in client cohort models

| **Model Outcome** | **Covariates included** |
| --- | --- |
| ART adherence | Clinic |
| Retention in care | Clinic |
| Quality of communication | Clinic, income |
| Participatory decision-making | Clinic |
| Satisfaction with HIV care | Clinic, gender |
| HIV stigma (overall) | Clinic, gender, years living with HIV |
| Anticipated HIV stigma | Clinic, years living with HIV |
| Enacted HIV stigma | Clinic, gender, years living with HIV |
| Internalized HIV stigma | Clinic, gender, years living with HIV |

Notes: We tested variables that differed at a level of p < 0.10 between treatment arms as covariates in the models testing intervention effect; only variables significant at p < 0.05 and/or making a difference in the intervention effect were maintained in the final models. All provider/staff models control for clinic, cadre, and gender, as they were relevant for all models. HIV client model adjustments vary by model based on covariates, and are listed by model in the table above.

# Table B. Statistics for the time by intervention by gender interaction on competence to provide gender-sensitive care among the HIV provider cohort

|  | *Arm*Time*Gender* | |
| --- | --- | --- |
|  | B (95% CI) | *p* |
| Intervention*12-months*Women | 0.17 (-0.10, 0.44) | 0.21 |
| Intervention*12-months*Men | 0.12 (-0.08, 0.32) | 0.24 |
| Intervention*6-months*Women | 0.30 (0.05, 0.54) | **0.02** |
| Intervention*6-months*Men | 0.37 (0.16, 0.59) | **<0.001** |
| Intervention*Baseline*Women | 0.07 (-0.14, 0.28) | 0.52 |
| Intervention*Baseline *Men (ref) |  |  |
| Control*12-months*Women | 0.17 (0.02, 0.32) | **0.03** |
| Control*12-months*Men (ref) |  |  |
| Control*6-months*Women | 0.03 (-0.10, 0.17) | 0.65 |
| Control*6-months*Men (ref) |  |  |
| Intervention*Baseline*Women (ref) |  |  |
| Intervention*Baseline *Men (ref) |  |  |

Abbreviations: B=unstandardized beta, CI=confidence interval, ref=reference group

Notes: The model compares the intervention effect over time between women and men provider. The model controls for clinic, cadre, and gender. Bold indicates statistical significance at p < 0.05

# Table C. Statistics for the time by intervention by cadre interaction on competence to provide gender-sensitive care among the HIV provider cohort

|  | *Arm*Time*Gender* | |
| --- | --- | --- |
|  | B (95% CI) | *p* |
| Intervention*12-months*Certified Health Workers | 0.04 (-0.17, 0.26) | 0.69 |
| Intervention*12-months*Lay Health Workers & Staff | 0.16 (0.02, 0.30) | **0.02** |
| Intervention*6-months* Certified Health Workers | 0.27 (0.07, 0.47) | **0.009** |
| Intervention*6-months* Lay Health Workers & Staff | 0.32 (0.18, 0.47) | **<0.001** |
| Intervention*Baseline* Certified Health Workers | 0.18 (-0.27, 0.40) | 0.09 ^t^ |
| Intervention*Baseline * Lay Health Workers & Staff (ref) |  |  |
| Control*12-months* Certified Health Workers | 0.15 (0.00, 0.31) | 0.05 ^t^ |
| Control*12-months* Lay Health Workers & Staff (ref) |  |  |
| Control*6-months* Certified Health Workers | -0.06 (-0.18, 0.06) | 0.35 |
| Control*6-months* Lay Health Workers & Staff (ref) |  |  |
| Intervention*Baseline* Certified Health Workers (ref) |  |  |
| Intervention*Baseline * Lay Health Workers & Staff (ref) |  |  |

Abbreviations: B=unstandardized beta, CI=confidence interval, ref=reference group

Notes: The model compares the intervention effect over time between two categories of cadre: certified health workers and lay health workers. The model controls for clinic, cadre, and gender. Bold indicates statistical significance at p < 0.05; ^t^ indicates p < 0.10.

# Figure A. Depiction of the time by intervention by gender interaction on perceived participatory decision-making with HIV providers among the client cohort

Notes: The figure depicts the intervention effect over time between women and men clients on the perceived level of participatory decision-making with HIV providers. The model controls for the following covariates: income, years living with HIV, clinic. Detailed statistics are provided in Table S4.

# Table D. Statistics for the time by intervention by gender interaction on perceived participatory decision-making with HIV providers among the client cohort

|  | *Arm*Time*Gender* | |
| --- | --- | --- |
|  | B (95% CI) | *p* |
| Intervention*12-months*Women | -0.40 (-0.84, 0.05) | 0.08 ^t^ |
| Intervention*12-months*Men | 0.16 (-0.25, 0.57) | 0.44 |
| Intervention*6-months*Women | -0.46 (-0.90, -0.02) | **0.04** |
| Intervention*6-months*Men | -0.003 (-0.44, 0.43) | 0.99 |
| Intervention*Baseline*Women | -0.34 (-0.71, 0.03) | 0.07 ^t^ |
| Intervention*Baseline *Men (ref) |  |  |
| Control*12-months*Women | -0.14 (-0.27, -0.00) | **0.04** |
| Control*12-months*Men (ref) |  |  |
| Control*6-months*Women | 0.37 (0.06, 0.68) | **0.02** |
| Control*6-months*Men (ref) |  |  |
| Control*Baseline*Women (ref) | -0.04 (-0.38, 0.31) | 0.84 |
| Control*Baseline *Men (ref) |  |  |

Abbreviations: B=unstandardized beta, CI=confidence interval, ref=reference group

Notes: The model compares the intervention effect over time between women and men clients on the perceived level of participatory decision-making with HIV providers. The model controls for the following covariates: gender, income, travel time to clinic, years living with HIV, clinic. Bold indicates statistical significance at p < 0.05, ^t^ indicates p < 0.10.

# Table E. CONSORT 2010 checklist of information to include when reporting a pilot or feasibility trial

| **Section/Topic** | **Item No** | **Checklist item** | **Reported on page No** |
| --- | --- | --- | --- |
| **Title and abstract** | | | |
|  | 1a | Identification as a pilot or feasibility randomised trial in the title | Title page |
|  | 1b | Structured summary of pilot trial design, methods, results, and conclusions (for specific guidance see CONSORT abstract extension for pilot trials) | Abstract, pg 1 |
| **Introduction** | | | |
| Background and objectives | 2a | Scientific background and explanation of rationale for future definitive trial, and reasons for randomised pilot trial | 2-5 |
|  | 2b | Specific objectives or research questions for pilot trial | 5 |
| **Methods** | | | |
| Trial design | 3a | Description of pilot trial design (such as parallel, factorial) including allocation ratio | 5-6 |
|  | 3b | Important changes to methods after pilot trial commencement (such as eligibility criteria), with reasons | N/A |
| Participants | 4a | Eligibility criteria for participants | 6-7 |
|  | 4b | Settings and locations where the data were collected | 6 |
|  | 4c | How participants were identified and consented | 7 |
| Interventions | 5 | The interventions for each group with sufficient details to allow replication, including how and when they were actually administered | Tables 1, pgs 8-13 |
| Outcomes | 6a | Completely defined prespecified assessments or measurements to address each pilot trial objective specified in 2b, including how and when they were assessed | Table 2 |
|  | 6b | Any changes to pilot trial assessments or measurements after the pilot trial commenced, with reasons | N/A |
|  | 6c | If applicable, prespecified criteria used to judge whether, or how, to proceed with future definitive trial | N/A, available in protocol paper |
| Sample size | 7a | Rationale for numbers in the pilot trial | 7-8 |
|  | 7b | When applicable, explanation of any interim analyses and stopping guidelines | N/A |
| Randomisation: |  |  |  |
| Sequence  generation | 8a | Method used to generate the random allocation sequence | N/A |
|  | 8b | Type of randomisation(s); details of any restriction (such as blocking and block size) | 6 |
| Allocation  concealment  mechanism | 9 | Mechanism used to implement the random allocation sequence (such as sequentially numbered containers), describing any steps taken to conceal the sequence until interventions were assigned | N/A |
| Implementation | 10 | Who generated the random allocation sequence, who enrolled participants, and who assigned participants to interventions | N/A |
| Blinding | 11a | If done, who was blinded after assignment to interventions (for example, participants, care providers, those assessing outcomes) and how | 7 |
|  | 11b | If relevant, description of the similarity of interventions | N/A |
| Statistical methods | 12 | Methods used to address each pilot trial objective whether qualitative or quantitative | 16-18 |
| **Results** | | | |
| Participant flow (a diagram is strongly recommended) | 13a | For each group, the numbers of participants who were approached and/or assessed for eligibility, randomly assigned, received intended treatment, and were assessed for each objective | Figure 2 |
|  | 13b | For each group, losses and exclusions after randomisation, together with reasons | Figure 2 |
| Recruitment | 14a | Dates defining the periods of recruitment and follow-up | 5 |
|  | 14b | Why the pilot trial ended or was stopped | N/A |
| Baseline data | 15 | A table showing baseline demographic and clinical characteristics for each group | Table 3 |
| Numbers analysed | 16 | For each objective, number of participants (denominator) included in each analysis. If relevant, these numbers should be by randomised group | Figure 2 |
| Outcomes and estimation | 17 | For each objective, results including expressions of uncertainty (such as 95% confidence interval) for any  estimates. If relevant, these results should be by randomised group | Tables 3 and 4 |
| Ancillary analyses | 18 | Results of any other analyses performed that could be used to inform the future definitive trial | Qualitative results, Table 5; Supplemental analyses |
| Harms | 19 | All important harms or unintended effects in each group (for specific guidance see CONSORT for harms) | N/A |
|  | 19a | If relevant, other important unintended consequences | N/A |
| **Discussion** | | | |
| Limitations | 20 | Pilot trial limitations, addressing sources of potential bias and remaining uncertainty about feasibility | 39-40 |
| Generalisability | 21 | Generalisability (applicability) of pilot trial methods and findings to future definitive trial and other studies | 39-40 |
| Interpretation | 22 | Interpretation consistent with pilot trial objectives and findings, balancing potential benefits and harms, and  considering other relevant evidence | 35-41 |
|  | 22a | Implications for progression from pilot to future definitive trial, including any proposed amendments | 35-41 |
| **Other information** | | |  |
| Registration | 23 | Registration number for pilot trial and name of trial registry | Abstract & 6 |
| Protocol | 24 | Where the pilot trial protocol can be accessed, if available | 6 |
| Funding | 25 | Sources of funding and other support (such as supply of drugs), role of funders | Title page & page 6 |
|  | 26 | Ethical approval or approval by research review committee, confirmed with reference number | 6 |
