# Supplementary material for: Mixed methods pilot evaluation of a gender-sensitivity training for HIV care providers in Uganda: Effects on providers and clients
Source: PLOS Glob Public Health. 2025 Sep 18;5(9):e0004247. doi: 10.1371/journal.pgph.0004247 (PMC12445538; doi:10.1371/journal.pgph.0004247)
Supplement: Attachment — Submitted filename: Response to PGPH.docx [file pgph.0004247.s012.docx]

Response to PGPH-D-24-02729 rejection

1. **Feedback from Editorial office:** Based on our evaluation of your manuscript and the information you provided, we do not feel that your submission meets our ethical requirements for human subjects research submissions. PLOS Global Public Health requires that research meets all applicable standards for the ethics of experimentation and research integrity (<http://journals.plos.org/globalpublichealth/s/human-subjects-research>). We reserve the right to reject any submission that does not meet our internal ethical standards, which in some cases are more stringent than local ethical standards.
   a. **Response:** This manuscript was previously submitted under PGPH-D-24-02729. The paper was rejected based on the ethics review board approval letter that was uploaded being incorrect. In the original submission, we accidentally submitted the approval letter for a different study. In this submission, we have corrected the file. The study was approved by both the Makerere University School of Public Health and University of Texas at San Antonio’s institutional review boards. Both letters are provided. We apologize for the error and thank you for your continued consideration of this manuscript.
