# Supplementary material for: Mixed methods pilot evaluation of a gender-sensitivity training for HIV care providers in Uganda: Effects on providers and clients
Source: PLOS Glob Public Health. 2025 Sep 18;5(9):e0004247. doi: 10.1371/journal.pgph.0004247 (PMC12445538; doi:10.1371/journal.pgph.0004247)
Supplement: Attachment — Submitted filename: PLOS Global Health Revision_Response to comments.docx [file pgph.0004247.s013.docx]

**PLOS One Global Health Revision**

Thank you to the reviewers and the editor for their helpful comments on our manuscript. We appreciate the time and effort put into the review process, and we believe your feedback has helped improve the clarity and quality of the work. In the following, we address each comment and outline the revisions made in response.

Reviewer #1: The nature of the study is quite interesting and relevant being a pilot study and issue of public health importance. It is also of note that pilot studies usually face challenges especially in terms of methodology and communication of findings. The authors did fairly well in overcoming the challenges. However, to make the manuscript worthy of publishing in line with standards of PLOS Public Health policies, the following weaknesses were noticed and should be addressed.

1. The authors are advised to study well the citation style of PLOS Public Health and adhere strictly, particularly in-text citations.
   a. **Response:** Following PLOS Global Health’s guidelines listed in the link below, we follow Vancouver style. This is also used in the in-text citations, following numbered style in brackets.
   b. <https://journals.plos.org/globalpublichealth/s/submission-guidelines>
2. The manuscript seem voluminous, hence, contains some sentences considered unnecessary and needless repetitions e.g. in Page 4, line 3 and 4, one sentence could have represented the two sentences on selected facilities.
   a. **Response:** We have reviewed the manuscript for areas to improve the precision of language, including the specific repetition noted by the reviewer. However, it is worth noting that mixed methods evaluations of interventions tend to be on the longer side, given the presentation of the two forms of data and the need to describe an intervention trial’s methods and specific intervention.
3. I could not find any mention of ethical permission which I consider necessary with the nature of the study.
   a. **Response:** This is on page 6. The details are masked for blind review but will be added if accepted for publication. IRBs both in Uganda and in the US approved the study. On page 7, we also specify that written informed consent was obtained from participants. IRB approval letters have been submitted per journal requirements as well, but this may not be available to reviewers.
      i. “The institutional review boards at the [MASKED FOR REVIEW] and the Ugandan National Council for Science and Technology, approved the study.”
4. Make sampling techniques and methodology in concise and familiar statements to make them reproducible for other intending researchers.
   a. **Response:** We have made revisions to the sampling techniques/methods on page 7 to include familiar statements to make the methods easy to identify, such as “We purposively sampled providers…”
5. There was no mention of what was done to minimize bias in the selection of respondents and this can lead to cofounders and/or chance.
   a. **Response:** The use of specific criteria to match clinics is described on page 7 – we have added additional text so that the relevance to bias reduction is apparent to the reader. We have also added the text below to page 8-9 specific to individual recruitment.
      i. “Health facilities were selected in consultation with Mildmay Uganda based on similarities in facility-level, demographics (e.g., semi-rural), number of ART clients, number of HIV care providers/staff, and HIV service implementation following MOH guidelines (immediate ART initiation, routine viral load monitoring), which were matched to minimize the risk of confounding variables.”
      ii. “The use of clear inclusion/exclusion criteria programmed into a computerized interviewer-administered questionnaire was used to reduce selection bias. “
6. Findings should be made concise and point-blank. Kindly report findings on quantitative and qualitative study separately.
   a. **Response:** We appreciate the reviewer’s suggestion to separate the quantitative and qualitative findings. However, presenting the results together is consistent with mixed methods research, which emphasizes the integration of both data types to provide a richer, more comprehensive understanding of the research problem. As Creswell and Plano Clark (2018) note, the integration of findings is a core principle of mixed methods, allowing for a more nuanced interpretation where qualitative data contextualizes and deepens the quantitative results.

Teddlie and Tashakkori (2009) highlight that combining both data forms enables triangulation, enhancing the validity of the study. Johnson, Onwuegbuzie, and Turner (2007) also emphasize the complementary nature of qualitative and quantitative data, where each provides insights that the other cannot fully capture. Presenting them together aligns with best practices in mixed methods research, as it fosters a more coherent narrative (Fetters, Curry, & Creswell, 2013). Thus, we believe that integrating the findings is a strength of this manuscript, enhancing its depth and clarity.

**References**:

Creswell, J. W., & Plano Clark, V. L. (2018). *Designing and Conducting Mixed Methods Research* (3rd ed.). Sage Publications.

Fetters, M. D., Curry, L. A., & Creswell, J. W. (2013). Achieving integration in mixed methods designs—principles and practices. *Health Services Research*, 48(6pt2), 2134-2156.

Johnson, R. B., Onwuegbuzie, A. J., & Turner, L. A. (2007). Toward a definition of mixed methods research. *Journal of Mixed Methods Research*, 1(2), 112-133.

Teddlie, C., & Tashakkori, A. (2009). *Foundations of Mixed Methods Research: Integrating Quantitative and Qualitative Approaches in the Social and Behavioral Sciences*. Sage Publications.

7. Improve in the use of punctuation in the entire manuscript e.g in Page 4, line 3 and 4, one study found health workers had explicit, negative....
   a. **Response**: We have reviewed the manuscript with a specific focus on punctuation. Below is the change made to the specific example pointed out by the reviewer. It now reads as follows:
      i. One study found health workers held negative bias towards men as HIV clients; they viewed them as “bad clients” based on assumptions about men being prideful and selfish and expressed little sympathy towards their healthcare engagement challenges.

Reviewer #2: General comments:
This manuscript presents the results from an gender-sensitivity training intervention for HIV care providers. The study was performed in six clinics in Uganda.

8. To set expectations, I am a quantitative researcher, so I will reserve comment on the qualitative aspects of this manuscript, mostly on the statistical methods. My read is that the statistical analyses focus around the use of regression models that utilize generalized estimating equations (GEE) to account for clinic-level clustering and repeated measures by participant.

   First, regarding the statistical power, clustering was not taken into account. For a group randomized trial (GRT), Murray et al (Am J Public Health, 2004;94:423) commented that, "...it is unusual for a GRT to have adequate power with fewer than 8 to 10 groups per condition."

   Second, GEE-based models are often referred to as population-averaged or marginal models. As such, they generally need quite a few clusters to ensure that the GEE approach is able to provide a valid marginal estimate. In the "Data analysis approach" subsection it is not stated what is used as the clustering variable. My assumption is that you have treated clinical as the cluster. In that case, there are far too few clusters for a marginal model. The Murray et al. article also quotes another article of their suggesting that GEE/sandwich estimator standard errors require 40+ total groups (20+ per arm in a balanced study). There are some small number of clusters corrections (e.g., Mancl-DeRouen is one I remember), but I am not sure if these are available in SPSS. Leyrat et al. (Intl J Epidemiol, 2018;47:321) concludes that the corrections are very important to correct the type I error rate, but are very underpowered.

   Thus, my major comment about your analyses is that I think this study is likely underpowered even with corrections. That is something that goes against criterion #3 in the PLOS ONE publication criteria (<https://journals.plos.org/plosone/s/criteria-for-publication>). I know that you have indicated in your language that this is a "pilot trial" and the results are "preliminary" and provide "promise". All of that concurs with the nature of this trial, but the quantitative methods chosen (especially GEE) do not concur with this.

   The most common alternative is to run a conditional model, usually a mixed model also known as a random effects (RE) model. The interpretation differs (it is at the individual level rather than the group level) but mixed models require fewer clusters because the results are at an individual level rather than a group level. Though, given Murray et al.'s comment about GRTs, you are also in a situation where you don't really have enough clusters for robust comparisons between arms. That's because the random intercepts per clinic are used to build a distribution, but it's hard to build a distribution with so few intercepts.

   Unfortunately, I think the analyses fall into that situation where you have too few clusters for REs and GEE, but enough that it feels necessary to account for clustering. I thought there was a paper that suggested including the clusters as fixed effects provides (roughly) the same coefficients and SEs as treating the clusters as random effects in a situation like this, but I am not able to find it at this time. Maybe I'm thinking of Abadie et al. (Quar J Economics, 2023;138:1. Regardless, that is my recommendation here is to include the clusters as fixed effects in a regression model and note in the limitation that there are not enough clusters to account for within-cluster correlation. Though, I am open to other suggestions.

- **Response:** We thank the reviewer for the thoughtful advice they’ve provided on our analytic approach. As the reviewer acknowledges, we are upfront in the manuscript about the limitations of the pilot trial in regards to power, and are therefore careful about the language we use, to not overstate the goals or conclusions made from such a trial. Nevertheless, we agree that we should use the analysis methodology that is most fitting for the data and can account for the clustered nature of the data as much as possible. As such, we appreciate the advice on the use of the regression model with the clusters included as fixed effects. We have fully rerun the models with this approach in this revision. However, we still use GEE, because the data is still repeated measures data, and must also account for having the same individual’s data at three time points. This new approach did not significantly change the results, but all results and relevant conclusions have been updated. Other relevant updates are displayed below:
- **Data analysis approach:** “Next, to evaluate the time by intervention effect on primary and secondary outcomes, we used Generalized Estimating Equations (GEE) in SPSS v. 28 to account for the repeated measures. We included clinic as a fixed effect to account for potential between-cluster variation in outcome measures. Given the limited number of clusters in this pilot trial, a random effects approach would not provide stable estimates; thus, treating clinic as a fixed effect allows for more reliable control of cluster-level confounding [48, 49].”
- **Limitations:** “Beyond the sample size limitations of a pilot trial, the pilot had few clusters, limiting our ability to fully account for within-cluster correlation in our analysis.”

References

48. Gelman A, Hill J. Data analysis using regression and multilevel/hierarchical models. Cambridge: Cambridge University Press; 2006.

49. Raudenbush SW, Bryk AS. Hierarchical linear models: Applications and data analysis methods. 2nd ed. Thousand Oaks: Sage Publications; 2002.

9. Another question I had is whether the intervention is given in small groups. In that case, you might have an individually randomized group treatment trial (IGRT). The key here is whether the intervention is delivered in small groups. I suggest reading this article <https://ajph.aphapublications.org/doi/full/10.2105/AJPH.2007.127027> to see if your design would qualify as an IGRT and adjust accordingly. I bring this up because the small groups may be a more accurate representation of the dependency between observations in these data. Also, it would give you more clusters and maybe GEE will work then and I've written this for nothing.
   a. **Response:** Thank you for this insight and for sharing Pal et al.’s article. People were trained in a group that generally aligned with the clinic they were in. There were only a total of 3 training cohorts, with some smaller clinics combined. As such, we have taken the first suggestion under comment 8.
10. (p.8) What is a "cell"?
    a. **Response:**
       i. It simply refers to group or condition; we have revised it to say condition.
11. Including line numbers greatly helps with the review process and I encourage you to submit papers with line numbers in the future.
    a. **Response**:
       i. Noted. We have added line numbers for the revision.
