# Supplementary material for: Mixed methods pilot evaluation of a gender-sensitivity training for HIV care providers in Uganda: Effects on providers and clients
Source: PLOS Glob Public Health. 2025 Sep 18;5(9):e0004247. doi: 10.1371/journal.pgph.0004247 (PMC12445538; doi:10.1371/journal.pgph.0004247)
Supplement: Attachment — Submitted filename: PLOS Global Public Health_Reponse to reviewers.docx [file pgph.0004247.s014.docx]

Thank you for taking the time to review our manuscript and provide such thoughtful and constructive feedback. We appreciate the reviewers’ careful consideration and insightful comments, which have significantly contributed to improving the clarity, rigor, and overall quality of the paper. We have carefully addressed each point raised and incorporated the suggested revisions, as detailed in the responses below.

**Reviewer #1**: The Authors have demonstrated desires to improve on their manuscripts as all issues raised in the previous revision have been addressed, I want to advise the Authors to make some minor improvements in the manuscript.

1. conjunction “And” appears to have been overused in line 85 of the manuscript
   a. **Response:** We have revised the sentence, removing one “and” – it reads as follows:
      i. “PEPFAR defines client-centered care as services that meet the needs of individuals by increasing convenience, providing welcoming services to diverse populations, making services supportive and responsive, and engaging communities and stakeholders.”
2. I’m of the view that statement in line 90 can be better written as “However there is a gap in researches that focus on building healthcare ……..” except if the Authors strongly believe otherwise.
   a. **Response**: We agree and have made this change.
      i. “However, there is a gap in research that focuses on building health care providers’ capacity…”

**Reviewer #2**: General comments:

This manuscript reports the results of an intervention focused on gender-sensitive HIV training implemented in Uganda using a mixed methods approach.

1. First off, I struggled with the cause and effect in this trial. Although randomization is performed at the clinic-level and the clinics are matched, the analyses are all at the patient level. Thus, there is no guarantee that the randomization will balance the intervention and control groups at the individual level. Indeed, you mention this in the limitations, but then there is nothing really done to address this, other than throwing variables in a regression model and hoping it works out. This is not the typical design since there is some randomization, and multiple sources of variability (clinic clusters, maybe the intervention group, longitudinal measurements). But I think more could be done to achieve a better balance between groups to provide a stronger causal effect. Invers
2. e probability of treatment weighting might be an option.
   a. **Response:** We appreciate the reviewer’s thoughtful feedback regarding the study design and interpretation. As a pilot trial, this study was designed primarily to assess feasibility, acceptability, and preliminary signals of effect, rather than to establish definitive causal conclusions. The limited number of clusters and modest sample size reflect the practical constraints typical of pilot work. Our goal was to explore whether the intervention may show promise in shifting trends — thus supporting the case for future, larger-scale testing or adaption before moving the intervention into a larger trial.

Importantly, we randomized at the clinic level (rather than the individual level) to avoid contamination between arms, which is a known risk in provider-targeted interventions. While this introduces some complexity in the analytic design, it also allowed us to test implementation procedures that would be applicable in a future full-scale cluster randomized controlled trial (CRCT) or stepped wedge design. We have been careful throughout the manuscript to align the paper’s aims and conclusions with those of a pilot trial, avoid overstating implications, and clearly acknowledge the limitations — including the inability to infer causality.

- “Given the strong covariate balance in the provider cohort and the small sample size, we did not apply inverse probability of treatment weighting, though we acknowledge it as an alternative approach in larger studies with greater imbalance.”

There is a growing body of work supporting the use of pilot and proof-of-concept trials in implementation research to refine intervention content and delivery before scaling up. We have cited below several key studies from the intervention and Implementation Science literature to support this rationale:

- Curran GM, Bauer M, Mittman B, Pyne JM, Stetler C. *Effectiveness-implementation hybrid designs: combining elements of clinical effectiveness and implementation research to enhance public health impact.* Med Care. 2012;50(3):217–26 z. zdoi:10.1097/MLR.0b013e3182408812
- Rounsaville BJ, Carroll KM, Onken LS. A stage model of behavioral therapies research: Getting started and moving on from stage I. Clin Psychol: Sci Practice. 2001;8(133-142).
- Eldridge SM, Chan CL, Campbell MJ, et al. *CONSORT 2010 statement: extension to randomized pilot and feasibility trials.* BMJ. 2016 Oct 24;355:i5239. doi:10.1136/bmj.i5239

We recognize the reviewer’s concern that relying solely on regression adjustment may not fully account for individual-level imbalance in a clustered design. We appreciate the suggestion to consider inverse probability of treatment weighting (IPTW) as an alternative approach to improve balance and strengthen causal inference. While IPTW can be valuable in certain contexts, particularly in observational studies with baseline imbalance, we opted not to use IPTW in this study for several reasons:

**i. Strong Covariate Balance in the Provider Cohort**. In our primary analysis cohort (N = 144 providers; 61 intervention, 83 control), we observed strong covariate balance between arms. Given this, we determined that standard regression-based adjustment was sufficient. We believe that IPTW was unlikely to materially alter the results or provide additional benefit in this context.

**ii. Sample Size Constraints**. The small size of the provider cohort limits the stability of IPTW. As noted in prior work, IPTW can generate extreme weights when estimated propensity scores approach 0 or 1, which can increase variance and reduce the precision of treatment effect estimates — particularly in small samples (Austin & Stuart, 2015). We were concerned that implementing IPTW would introduce analytic noise rather than clarity.

**iii. Indirect Exposure in the Patient Cohort**. Although greater imbalance was observed in the patient cohort (N = 238; 119 per arm), these individuals were not directly randomized to receive the intervention. Their outcomes are only indirectly affected through provider behavior. Applying IPTW at the patient level would therefore not reflect a direct causal contrast and may limit interpretability. For this reason, we did not pursue IPTW in the patient analysis either.

Given these considerations, we focused on traditional regression adjustment and transparent reporting of baseline covariates and outcomes. We have now added a note in the Discussion section acknowledging IPTW as a potential analytic strategy and explaining our rationale for not applying it in this pilot setting.

**Reference:**
Austin PC, Stuart EA. *Moving towards best practice when using inverse probability of treatment weighting (IPTW) using the propensity score to estimate causal treatment effects in observational studies.* Stat Med. 2015;34(28):3661–79. doi:10.1002/sim.6607

3. Also, it seemed as though the intervention was applied in small groups. I couldn't tell if people stayed in the same group. If so, that could be another source of variability since there could be correlation between members of the same group.
   a. **Response:** People were trained in a group that generally aligned with the clinic they were in. There were only a total of 3 training cohorts, with some smaller clinics combined. We have added this to the methods section, as follows:
      i. “There were only a total of 3 training cohorts in the intervention arm (with people’s cohort generally aligning with their clinic).”
4. I also wondered about how useful this study is a pilot, based on the limitations mentioned in lines 703-706. You mentioned that an efficacy trial will collect viral load from patients and not use self-report measures. Your primary aim (line 142) seems to be to gather preliminary evidence. Not collecting data with the outcome that would be used in the trial seems like a big shortcoming. Do you have information on how reliable self-report data are?
   a. **Response:** We appreciate the reviewer’s important observation regarding the use of self-reported adherence rather than viral load as the primary outcome in this pilot study. While we agree that biologic outcomes such as viral load will be critical for a future efficacy trial, the use of self-report in this pilot was intentional and aligned with the study’s goals, scope, and resource constraints.

First, the primary aim of this pilot was to assess feasibility, acceptability, and preliminary signals of intervention effect — not to evaluate efficacy per se. Given the limited scope and budget typical of pilot trials, collecting viral load data was not feasible across the study population. Instead, we focused on measures that are validated, cost-efficient, and capable of capturing early trends in behavior change.

To that end, we used the 4-day adherence recall from the Adult AIDS Clinical Trials Group (AACTG) scale [53], a widely used and validated tool. This measure has demonstrated strong construct validity in Uganda [54] and has shown significant correlations with viral load in multiple settings [55], supporting its use as a proxy for medication adherence in contexts where biologic measurement is not feasible. This information is provided in Table 2.

Moreover, this study also collected data on other components of the conceptual model (see Table 2), along with qualitative data to help triangulate findings and inform intervention refinement for the next phase of research.

I had many comments on analytic aspects of the manuscript. Those are below in the specific comments section.

5. (lines 43,52 and throughout the manuscript) I'd recommend shifting away from test statistics and p-values and reporting the point estimate and confidence interval (CI). That conveys more information to readers. In addition, it concurs with the shift away from p-values.
   a. **Response:** Thank you for this suggestion. We have added the CIs throughout as requested.
6. (lines 46-47, 353-354, and throughout the manuscript) Same theme as my previous comment; it's more useful to have the CI rather than a standard error and p-value.
   a. **Response:** See response to comment 4.
7. (line 212) Citation needed for G * Power. Always include the version of any software used.
   a. **Response:** Thank you for pointing this out. We have added the citation and version. The version was 3.1.

Faul, F., Erdfelder, E., Buchner, A., & Lang, A. G. (2009). Statistical power analyses using G*Power 3.1: tests for correlation and regression analyses. *Behavior research methods*, *41*(4), 1149–1160. https://doi.org/10.3758/BRM.41.4.1149

8. lines 205-216) I was confused by the statement in lines 206-208 that is quoting 15-30 per condition and then the rest of the paragraph which computes a power of 100 per condition. What point is being made by having the statement in lines 206-208?
   a. **Response:** We appreciate the reviewer’s question and recognize that the original paragraph may have lacked clarity. The point being made is that this was a pilot study, and as such, we followed standard recommendations for Stage 1b pilot trials (typically 15–30 participants per condition) when determining the minimum sample size for the provider cohort, where the intervention was delivered directly.

However, we also included a secondary client cohort, where the intervention effect would be indirect (i.e., through their providers). Because of this indirect effect, we aimed to recruit a larger sample in the client group than what is typically needed in pilot studies. This required a more formal a priori power estimation to ensure the sample could detect a moderate effect size on the client outcome (ART adherence), which was our main exploratory outcome for that group.

We have revised the manuscript text to make this rationale more transparent. The revised paragraph now reads:

- “As a pilot trial with the exploratory aim of examining the intervention’s preliminary effects, our sample size is based on guidelines for Stage 1b studies (pilot testing of new behavioral interventions), which suggest 15–30 participants per condition [37]. We aimed to recruit the full population of HIV care providers/staff per clinic, which was estimated to be ~20–35 providers per clinic and aligned with these recommendations. For the client cohort, where the intervention effect was expected to be indirect, we predefined a larger target sample of 120 clients per arm. We used G*Power (v.3.1) [40] to estimate that a final sample of 100 clients per condition (accounting for 17% attrition from n=120) would provide 80% power (α = 0.05) to detect a moderate effect size (d = 0.4) in ART adherence, consistent with similar adherence interventions in sub-Saharan Africa [41].”

9. (line 339) I don't know what are "generalized linear equations" are. Do you mean generalized linear models? I strongly, strongly recommend to always have methodological citations for your statistical methods. That way if this terminology is domain-specific (or misused) then a reader has a much better chance of determining the methods used.
   a. **Response:** Thank you for this helpful comment. You are absolutely correct — the original text should have referred to generalized linear models rather than “generalized linear equations,” and we appreciate your close reading in pointing this out.

We have revised the manuscript accordingly to ensure the correct terminology is used. In addition, we have now added a methodological citation to clarify the statistical framework employed in our analysis. The revised sentence in the Methods section now reads:

"We used generalized linear models [49] in SPSS v. 28 [50] to examine associations between study arm and primary outcomes, accounting for clustering at the clinic level."

We have also included the following reference in the reference list:

- McCullagh, P., & Nelder, J. A. (1989). *Generalized Linear Models* (2nd ed.). Chapman & Hall/CRC.

We appreciate your recommendation to include methodological references and have reviewed the rest of the methods section to ensure proper citation of other analytic techniques as well.

10. (lines 341-342) I know that people like to say they used GEE in methods sections, but unfortunately GEE is an optimization method and not a statistical method. GEE could be used along with many different regression-type methods. I presume you have used logistic regression with GEE to account for repeated measures. Please confirm this and include this information in your revisions.
    a. **Response:** Thank you for this clarification. You are absolutely correct that GEE is an optimization technique rather than a standalone statistical method, and we appreciate your careful attention to the distinction.

As noted in our response to Comment 10, the reference to GEE in the Methods section was inadvertently carried over from a previous version of the manuscript. In our initial submission, we had indeed used logistic/linear regression (depending on the outcome) with GEE to account for repeated measures. However, based on reviewer feedback regarding the small number of clusters, we fully revised our analytic approach for this submission. In the final analysis, we used generalized linear models with **clinic included as a fixed effect,** and did **not** use GEE. See comment 10 for more explanation.

We have corrected the manuscript to remove the reference to GEE and to clearly describe the analytic approach actually used in this version.

11. (lines 343-344) I don't disagree with what you have done by treating clinic as a fixed effect, though I don't understand what is being said here. It's impossible to include both random effects and GEE in the same model since they do not use the same optimization method. Given that is impossible, what are you arguing? Random effects are generally better than GEE-based approaches when there are a small number of clusters since there is good evidence that the sandwich estimator that is used in GEE is biased when the number of clusters is less than 40 (see Emrich and Piedmonte, J Stat Computation Simulation. 1992;41:19, Lipsitz et al, Biometrics. 1994;50:270, Feng et al, Stat Med. 1996;15:1793, Murray et al, Eval Rev. 1996;20:313). That bias is downward, meaning the confidence intervals are too small. Thus, a random effects model would be a better option with a small number of clusters. Are you arguing against a multilevel model with patients (and their longitudinal measurements) nested within clinics? Usually random effects models start having a chance to work when there are >=6 clusters, which is what you have. It's also important to note that the interpretations are different so it's not a direct swap between the two methods, but it's close.
    a. **Response:** Thank you for this insightful and technically important comment. You are absolutely right — our mention of generalized estimating equations (GEE) in the text was an error, inadvertently carried over from an earlier version of the manuscript. In our original submission, we used GEE to account for repeated measures. However, in response to comments from the first round of peer review — specifically related to the small number of clusters — we fully revised the analytic approach to use general regression models with clinic treated as a fixed effect, and did not use GEE or random effects in the final analysis.

We agree with your point that GEE is not ideal with a small number of clusters due to the potential downward bias of the sandwich estimator, as noted in the references you helpfully provided. While a random effects model (i.e., a multilevel model) may be preferable in many small-cluster settings, we opted to use fixed effects for clinic in this pilot study to conservatively adjust for between-clinic differences while avoiding the instability that can occur with random effects estimation when the number of clusters is limited.

We also recognize that a random effects model would provide a different interpretation — focusing on population-averaged versus cluster-specific effects — and appreciate the reminder that these are not interchangeable. We've revised the manuscript to remove the incorrect GEE reference and clarify that we are using fixed effects for clinic in the final models.

12. (Lines 345-347) In regards to variable selection, this approach is sometimes referred to as "bivariate screening". This approach has been criticized in literature (see Harrell, Jr. _Regression Modeling Strategies_. New York: Springer; 2001. and Wiegand, _Statist Med_, 2010;29:1647 <https://doi.org/10.1002/sim.3943>) and an example has been shown to miss important variables (Sun et al., _J Clin Epidemiol_, 1996;49:907 [http://dx.doi.org/10.1016/0895-4356\(96\)00025-X](http://dx.doi.org/10.1016/0895-4356%5C(96%5C)00025-X)). Regardless, the results from one of those studies (Wiegand, _Statist Med_, 2010;29:1647) suggested that at sample sizes of 1,000 or greater, stepwise variable selection procedures may do adequately well, especially when used with a larger p-value than 0.05. Sun et al. found that bivariate screening can miss a variable that may be a confounder even when a p-value higher than what you have used. Bivariate screening can be considered a form of stepwise variable selection, which usually does not do a good job of finding the most appropriate model (e.g., [[https://doi.org/10.1002/sim.3943](https://doi.org/10.1002/sim.3943)](https://doi.org/10.1002/sim.3943%5D(https:/doi.org/10.1002/sim.3943))). Generally it's better to select based on more robust criteria. I prefer shrinkage-based models, such as lasso, for variable selection. At a minimum, I would encourage using measures which assess the fit of the model. AIC or BIC may be possible, for which you could you Burnham and Anderson (doi: 10.1177/0049124104268644) as a guide.
    a. **Response:** Thank you for this thoughtful and well-supported comment. We agree that bivariate screening and stepwise approaches can be problematic, especially when used for confirmatory modeling or causal inference, and we appreciate your pointing us to key literature on this topic.

In our case, the use of bivariate associations was not intended as a stepwise model-building process or for confounder selection, but rather as a descriptive exploration to identify candidate variables that may warrant inclusion in future fully powered models. As this is a pilot study with a relatively small sample, our goal was to identify preliminary signals of association, recognizing that we are underpowered for complex model selection procedures. We have revised the manuscript to clarify this intent and to ensure we do not overstate the role of the bivariate screening step (see new text in methods section below).

In response to your recommendation, we also conducted a sensitivity analysis comparing model fit using Akaike Information Criterion (AIC) for alternative specifications. The results were consistent (not reported), but we acknowledge that any model selection in this pilot context should be interpreted with caution and primarily used to inform design and power estimation in future trials.

- “We explored baseline differences between treatment arms and included variables that differed at a level of *p* < 0.10 as covariates in preliminary models testing intervention effects. This approach was used to identify potential imbalances and inform model specification in this exploratory, pilot context. Final models retained covariates based on their theoretical relevance, observed association with outcomes, and/or meaningful influence on the intervention effect estimate, rather than solely on statistical significance.”

13. (Line 688) In this line you talk about effectiveness but then later in the paragraph you mention doing a community efficacy trial. Which is it? Efficacy is the effect of an intervention under ideal circumstances and effectiveness is the effect of an intervention in real-world practice.
    a. **Response:** Thank you for pointing this out. You are absolutely right to highlight the important distinction between efficacy and effectiveness. In our case, the intervention is being tested for the first time, so conceptually this aligns with an early-stage (efficacy-focused) evaluation. However, because the intervention is implemented under real-world conditions — using local implementers, in existing health system settings, with researcher control but not as strict as more controlled trials — the trial (and the next future trial) also shares characteristics of an effectiveness study.

Given this blend, we have revised the text about future trials to specify effectiveness-implementation hybrid trial, Type 1 (Curran et al., 2012), which allows for the testing of a new intervention’s clinical outcomes while also gathering information on real-world delivery. To avoid confusion, we have revised the text to remove the phrase "community efficacy trial" and instead refer to the next step as a fully-powered hybrid effectiveness-implementation trial, which more accurately reflects the design.

14. (Line 696) Why are you saying there are only two clusters? I thought there were six clinics and those were the clusters.
    a. **Response:** Thank you for this helpful comment. You are absolutely right that six individual clinics participated in the study. The reference to "two clusters" reflects how the trial was originally designed and implemented, particularly given the constraints of a **pilot study**.

Specifically, the trial was originally conceptualized as a **two-site, quasi-experimental controlled pilot**, with one intervention clinic and one control clinic, each serving as a cluster. To meet recruitment goals — particularly for the client cohort — we expanded the study after launch to include two additional, smaller clinics in each arm. These added clinics were selected based on geographic proximity, demographic similarity, and comparable HIV care practices. Because they functioned as **extensions of the original sites**, and not independently randomized units, we treated each set of three clinics (per arm) as a **single implementation cluster.**

However, we recognize that, analytically, treating all six clinics as interchangeable units would oversimplify the structure. To address this, our **analysis includes clinic as a fixed effect**, allowing us to account for any between-clinic differences while preserving the original design intent. In other words, while the trial was designed and implemented as two matched clusters, the analytic model accounts for the **individual clinic-level variability**.

We previously described this in the methods section, but your comment has highlighted the need for revisions to clarify this distinction and to make it explicit that this structure reflects the **feasibility constraints and pragmatic goals** of a pilot trial. The new text reads as follows:

- “Health facilities were selected in consultation with Mildmay Uganda based on similarities in facility characteristics (e.g., semi-rural setting), number of ART clients, number of HIV care providers/staff, and HIV service delivery aligned with MOH guidelines (e.g., immediate ART initiation, routine viral load monitoring). The study was originally designed as a two-site, quasi-experimental pilot trial. Two hospitals — one per arm — were initially selected and randomly allocated via coin toss to receive either the intervention or serve as control. After recruitment began, to help meet targets for the HIV client cohort, two smaller clinics were added to each study arm. These additional clinics were selected using the same matching criteria and were located within the same geographic districts as the original sites. They were treated as extensions of the original facilities for implementation purposes. To reduce the risk of cross-contamination, clinics in the intervention and control arms were located more than one hour apart, minimizing the likelihood of clients or providers crossing between sites.”
