# Supplementary material for: Mixed methods pilot evaluation of a gender-sensitivity training for HIV care providers in Uganda: Effects on providers and clients
Source: PLOS Glob Public Health. 2025 Sep 18;5(9):e0004247. doi: 10.1371/journal.pgph.0004247 (PMC12445538; doi:10.1371/journal.pgph.0004247)
Supplement: Attachment — Submitted filename: Respomse to reviewers.docx [file pgph.0004247.s015.docx]

We would like to thank the reviewers and editors for their additional review and comments of this manuscript, as well as the quick turnaround in their response. We were pleased to learn that the two reviewers felt our previous revision had addressed all of their comments. The Editor has provided additional comments related to copy-editing and the over-interpretation of p-values. We appreciate these comments and have addressed them directly below and in the revised manuscript.

Editor Comments:

1. Results paragraph on baseline imbalance - should that be ‘treatment arms differed at baseline (p<0.05)’ not p<0.50?
   a. **Response:** Thank you for catching this error. Yes, it should be 0.05. We have made this change. It now reads as: “…treatment arms differed at baseline (p < 0.05).”
2. Please consider providing the units for the Beta values in this paragraph - years for HIV status and ART use, thousands of shillings for income? Hours for travel time?
   a. **Response:** We have added the units as recommended to this paragraph. It reads as follows:
      i. “Details are in Table 3, but in summary, clients from intervention clinics, on average, knew their status for **fewer years** (B = -2.70, 95% CI = -4.83, -0.56, p = 0.01) and were on ART for **fewer years** than those from control clinics (B = -1.88, 95% CI = -3.77, 0.02, p = 0.05). Intervention clients reported more hazardous alcohol use (OR = 2.63, 95% CI = 0.74, 4.51, p = 0.006), food insecurity (B = 0.07, 95% CI = 0.22, 0.36, p = 0.64), lifetime experience of intimate partner violence (OR = 2.81, 95% CI = 1.28, 6.16, p = 0.01), higher monthly income **in units of 40,000 UGX** (~10 USD) (B = 2.63, 95% CI = 0.74, 4.51, p = 0.006), and less travel time to the HIV clinic than the control **in units of 30 minutes** (B = -0.91, 95% CI = -1.32, 0.51, p < 0.001).”
3. Above Table 2- extraneous ‘)’
   a. **Response**: Thank you for catching this error. After the sentence pasted below, there was an unnecessary ‘)’ which has been removed.
      i. “As detailed in Table 2, measures to examine change in the intervention’s hypothesized primary and secondary outcomes for the HIV provider and client cohorts were collected, along with additional validated measures for potential covariates relevant for HIV care engagement outcomes.”
4. Results Thematic Area 2, next page ‘clients in both treatment arms’ == ‘clients in both arms’ since there is 1 intervention and 1 control arm?
   a. **Response:** Yes, thank you for this correction. We have changed it to “clients in both arms” and removed “treatment.” We searched the document and found a few other instances of this and corrected them.
5. Thematic area 3 quantitative results on emotional and stress regulation - ‘simple’ is non-specific, but presumably means unadjusted or crude? In addition, p values do not trend, they simply are or are not less than a given threshold. Please revise to state the results directly, e.g. self-reported use of regulation techniques was higher in the intervention than control arm at 6 months (B=0.42, …) and 12 months (B=0.30….)
   a. **Response**: This has been corrected to remove the use of the word “simple” as well as “trending.”
      i. “The reported use of these techniques increased at a level of statistical significance in intervention providers compared to control providers over time overall (Wald *χ^2^* = 8.17; p = 0.01) and at 6-months (6 months: B = 0.42, 95% CI = 0.13, 0.72, p = 0.005; 12-months: B = 0.30, 95% CI = -0.01, 0.61, p = 0.05).”
6. Similarly in Thematic Area 4 in the paragraph on stigma, the phrasing around trends introduces confusion as to whether the statistical test is an actual test of trends vs. the analysis as described that compares 6 month to baseline and 12 month to baseline. Preferable to report the betas and CIs and note if needed the effects with p<0.05.
   a. **Response**: We have revised this paragraph to remove “trending” and instead discuss directionality of change with reported betas and CIs. While we still report the p-value for all statistics, we have reduced the amount we rely on the p-value for interpretation.
      i. “In the client cohort followed over 12-months, ART adherence (main outcome) improved over time in both study arms with no difference between the two (Wald *χ^2^* = 2.53, p = 0.28; 6 months: B = 0.39, 95% CI = -6.67, 7.45, p = 0.91; 12-months: B = -3.05, 95% CI = -9.21, 3.11, p = 0.33). Adherence to HIV clinic appointments followed the same pattern (Wald *χ^2^* = 1.68, p = 0.43; 6 months: B = -0.01, 95% CI = -0.04, 0.03, p = 0.86; 12-months: B = -0.02, 95% CI = -0.05, 0.02, p = 0.32). Clients’ self-reported satisfaction with HIV care was static in the control clinics overtime with a modest reduction in intervention clinic clients, particularly between 6- and 12-months (Wald *χ^2^* = 15.04, p < 0.001; 6 months: B = -0.01, 95% CI = 0.18, 0.16, p = 0.91; 12-months: B = -0.29, 95% CI = -0.46, -0.13, p < 0.001). In favor of the intervention, clients in the training clinics reported a reduction in HIV stigma over time compared to clients in the control clinics (where an increase was observed) (Wald *χ^2^* = 18.72, p < 0.001; 6 months: B = 0.03, 95% CI = -0.14, 0.19, p = 0.76; 12-months: B = -0.28, 95% CI = -0.44, -0.13, p < 0.001). Examining the subscales that make up the overall HIV stigma score, the intervention arm’s reduction in HIV stigma was driven by reduced anticipated HIV stigma (Wald *χ^2^* = 23.84, p < 0.001; 6 months: B = 0.12, 95% CI = -0.14, 0.37, p = 0.37; 12-months: B = -0.46, 95% CI = -0.71, -0.22, p < 0.001). The other two stigma subscales were also reduced in the intervention compared to control, but not at a level of statistical significance (enacted HIV stigma: Wald *χ^2^* = 4.08, p = 0.13; 6 months: B = -0.06, 95% CI = -0.21, 0.09, p = 0.45; 12-months: B = -0.14, 95% CI = -0.29, 0.01, p = 0.06; internalized stigma: Wald *χ^2^* = 5.03, p = 0.08; 6 months: B = 0.02, 95% CI = -0.27, 0.32, p = 0.88; 12-months: B = -0.22, 95% CI = -0.45, 0.01, p = 0.06).”
   b. With the Editor’s comments in mind about the over-reliance of p-values, we have also reviewed the results section beyond the above 2 mentioned sections and made similar edits. For example, we removed similar “simple effects” language and reference to p-values under Thematic Area 1 (page 18).
